# Allogeneic Umbilical Cord Blood Serum Eyedrops for the Treatment of Severe Dry Eye Disease Patients

**DOI:** 10.3390/ijms262110782

**Published:** 2025-11-06

**Authors:** Marco Zeppieri, Giuseppe Gagliano, Matteo Capobianco, Caterina Gagliano, Francesco Cappellani, Giuseppa Tancredi, Alessandro Avitabile, Ludovica Cannizzaro, Fabiana D’Esposito

**Affiliations:** 1 Department of Ophthalmology, University Hospital of Udine, 33100 Udine, Italy; 2 Department of Medicine, Surgery and Health Sciences, University of Trieste, 34127 Trieste, Italy; 3 Department of Ophthalmology, University of Catania, 95123 Catania, Italy; 4 Department of Medicine and Surgery, University of Enna “Kore”, Piazza dell’Università, 94100 Enna, Italy; 5 Eye Center “G.B. Morgagni-DSV”, 95125 Catania, Italy; 6 Sciacca Cord Blood Bank, 92019 Sciacca, Italy; 7 Neurovisual Science Technology (NEST) srl, 95123 Catania, Italy; 8 Imperial College Ophthalmic Research Group (ICORG) Unit, Imperial College, London NW1 5QH, UK

**Keywords:** allogeneic cord blood serum, severe dry eye disease, filamentary keratitis, graft-versus-host disease, neurotrophic keratitis, Stevens-Johnson syndrome, regenerative medicine, advanced therapies for dry eye

## Abstract

Human allogeneic umbilical cord blood serum stands out as a potent adjunct to conventional therapies for ocular surface disorders related to severe Dry Eye Disease. By expediting ocular surface regeneration and fostering epithelial integrity, umbilical cord blood serum not only enhances subjective patient experiences but also improves objective clinical indicators. This makes it particularly useful in patients with corneal ulcers through ocular surface regeneration and anti-inflammatory activity. This retrospective, interventional, non-randomized clinical study aims to explore the efficacy of allogenic umbilical cord blood serum in patients who had previously received other treatments unsuccessfully. This study was a retrospective, non-comparative, interventional clinical study involving 55 patients (35 females and 20 males) aged 18–82 years with severe Dry Eye Disease who were unresponsive to standard treatments. The study was conducted at Eye Center “G.B. Morgagni-DSV”, Catania, Italy. Patients were categorized based on the etiology of severe Dry Eye Disease into four groups: group I consisted of 26 patients with filamentary keratitis and corneal ulcers associated with rheumatologic diseases such as Sjogren’s syndrome and systemic sclerosis; group II comprised 15 patients with graft-versus-host disease; group III consisted of 10 patients with corneal neurotrophic ulcers; group IV included four patients with Steven–Johnson syndrome. Outcomes evaluated before and after treatment were OSDI (Ocular Surface Disease Index) and SANDE (Symptom Assessment in Dry Eye) Questionnaires, VAS (Visual Analog Scale), Slit-Lamp Examination, Esthesiometry, Lissamine Green Staining, NIBUT (Non-Invasive Break-Up Time) and BUT, Fluorescein Staining with Photography and Oxford Classification, Schirmer Test, Best-Corrected Visual Acuity (BCVA), Meibography. We observed a significant improvement in SANDE, VAS and OSDI questionnaires, Schirmer Test, BUT, BCVA, and Oxford classification after treatment with allogeneic cord blood serum eyedrops. Clinical variables, such as corneal inflammation, conjunctivalization, corneal neovascularization, or pain, were also considered individually. Nevertheless, pain and inflammation reduced markedly over time until completely healed in all cases. Our study highlights the remarkable efficacy of allogeneic cord blood serum eyedrops in patients with severe Dry Eye Disease who have shown absent or inadequate response to usual treatments for dry eye. This underscores the need for further comprehensive investigations in this field.

## 1. Introduction

The ocular surface is a finely balanced system that safeguards the globe and sustains visual clarity. It comprises the corneal and conjunctival epithelium and a tri-layer tear film whose components act in concert as both a physical and biochemical barrier. The mucin layer, secreted by conjunctival goblet cells, promotes uniform tear spread and traps debris; the aqueous layer, produced by the lacrimal glands, supplies oxygen, electrolytes, nutrients, and antimicrobial factors; and the lipid layer, derived from the meibomian glands, limits evaporation and stabilizes the film during and between blinks. Failure in any one layer propagates to the entire system, producing tear film instability, increased friction at the epithelium, fluctuating optical quality, and—when persistent—epithelial injury with downstream inflammation and nociceptive/neuropathic pain [1,2].

Dry Eye Disease (DED) represents the clinical expression of this breakdown in homeostasis. The TFOS DEWS II consensus defines DED as a multifactorial disease of the ocular surface characterized by loss of tear film homeostasis, accompanied by symptoms, with etiologic roles for tear film instability and hyperosmolarity, ocular surface inflammation and damage, and neurosensory abnormalities [1]. In practice, the phenotype spans from episodic discomfort to severe, chronic disease with persistent epithelial defects, recurrent infection, or stromal scarring that threatens vision. Patients commonly report a gritty or burning sensation, fluctuating blurred vision, photophobia, and visual fatigue—symptoms that meaningfully erode daily functioning and quality of life [3].

From a population standpoint, DED is highly prevalent worldwide and remains a leading reason for ophthalmic consultations [4]. Point estimates vary with diagnostic criteria and sampling frames (≈5% to >50%), but consistent signals include higher prevalence in women and older adults—attributable in part to hormonal influences and the burden of autoimmune disease—and an emerging contribution from lifestyle factors such as prolonged screen exposure in younger individuals [5,6]. Together, these trends underscore DED as a growing public health concern with substantial personal and societal costs.

### 1.1. Current Treatments and Unmet Needs

Management follows a stepwise framework: patient education and environmental modification, regular use of preservative-free lubricants, and escalation to anti-inflammatory therapy and immunomodulators such as cyclosporine or tacrolimus; in advanced disease, punctal occlusion and therapeutic contact lenses are frequently required [3,7]. Despite this armamentarium, a subset of patients—those with systemic autoimmunity (e.g., Sjögren’s, systemic sclerosis), ocular graft-versus-host disease (GVHD), neurotrophic keratitis, or Stevens–Johnson syndrome (SJS)—remains difficult to stabilize. In these contexts, persistent epithelial breakdown and poorly controlled inflammation perpetuate pain, photophobia, and functional loss and carry a real risk of irreversible corneal damage [3,8].

### 1.2. Blood-Derived Therapies and the Role of UCBS

To bridge this gap, attention has turned to blood-derived therapies, which deliver epitheliotrophic and anti-inflammatory factors intrinsic to ocular surface repair. Autologous serum is the most established option and can improve signs and symptoms by supporting epithelial proliferation/migration and modulating surface inflammation [9]. Practical and biological limitations, however, are non-trivial: repeated venipuncture is required, and in patients with active systemic autoimmunity, pro-inflammatory mediators present in autologous preparations may blunt local benefit [10,11].

Umbilical cord blood serum (UCBS) has therefore emerged as an attractive alternative. Collected safely at birth, UCBS is enriched in epidermal growth factor (EGF), nerve growth factor (NGF), transforming growth factor-β (TGF-β), and vascular endothelial growth factor (VEGF), and it displays a cytokine milieu that favors tissue repair and immune modulation [12,13,14]. Mechanistically, these factors map onto clinical priorities in advanced ocular surface disease: EGF and TGF-β support epithelial closure and matrix remodeling, NGF addresses denervation-related healing deficits, and a predominantly anti-inflammatory profile can help extinguish chronic surface inflammation. Clinically, prior studies suggest benefits in severe DED—including Sjögren’s and ocular GVHD—and in some settings, UCBS may outperform autologous serum on key outcomes such as corneal staining and goblet-cell metrics [15,16,17].

### 1.3. Purpose of the Study

Despite encouraging evidence, UCBS use remains heterogeneous across centers, with variability in donor screening, processing, concentration, dosing frequency, and treatment duration. This study was designed to evaluate the efficacy and safety of UCBS eyedrops in a well-characterized cohort of patients with severe DED and complex ocular surface disease who had failed conventional therapy. Specifically, we aimed to (1) quantify changes in patient-reported symptoms using OSDI, SANDE, and VAS; (2) assess objective parameters of tear film stability, epithelial integrity, and visual function; and (3) explore differential responses across etiologic subgroups to clarify which patients are most likely to benefit from UCBS [18].

## 2. Results

### 2.1. Patient Population and Baseline Characteristics

Fifty-five patients (35 women, 20 men; 18–82 years) with severe DED or complex ocular surface disease refractory to prior therapy were included. Groups: rheumatologic (n = 26), GVHD (n = 15), neurotrophic ulcers (n = 10), SJS (n = 4). At baseline, all exhibited advanced disease: Oxford grade IV–V, markedly reduced TBUT, and high OSDI/SANDE scores. Baseline characteristics are summarized in Table 1.

### 2.2. Symptom Improvement

**Figure 1:**
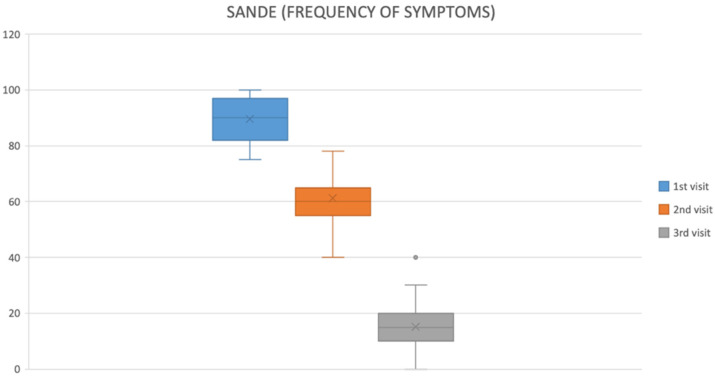
SANDE questionnaire (frequency of symptoms).

**Figure 2:**
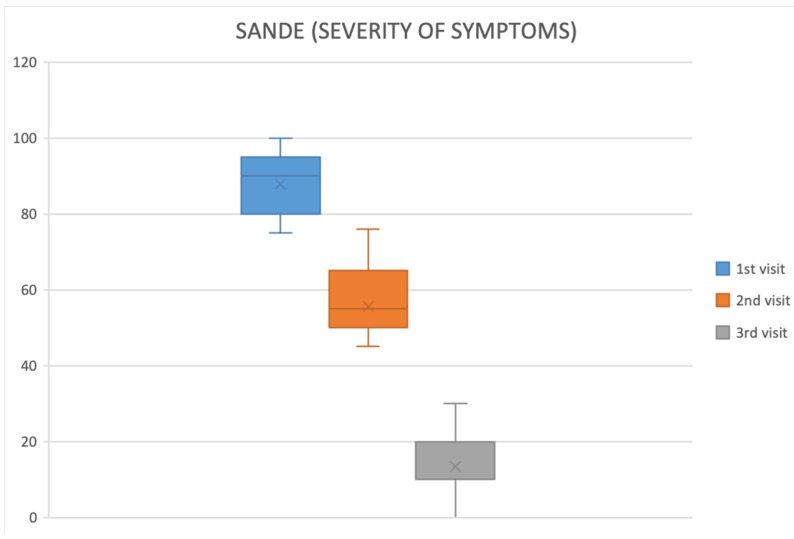
SANDE questionnaire (severity of symptoms).

**Figure 3:**
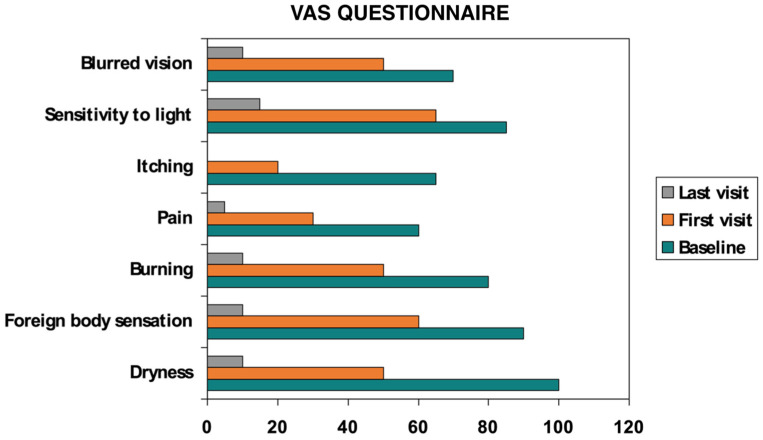
VAS questionnaire.

UCBS eyedrops led to clear, progressive symptom relief (Table 2):

- OSDI: 89.57 ± 7.79 at baseline → 61.22 ± 8.63 at first follow-up → 15.22 ± 11.33 at final visit (*p* < 0.05).
- SANDE: frequency and severity declined steadily across visits (Figure 1 and Figure 2).
- VAS: discomfort scores fell significantly from baseline to final evaluation (*p* < 0.05) (Figure 3).
- Improvements emerged earliest and most prominently in autoimmune and GVHD cohorts.

### 2.3. Objective Clinical Outcomes

#### 2.3.1. Tear Film Stability and Production

**Figure 4:**
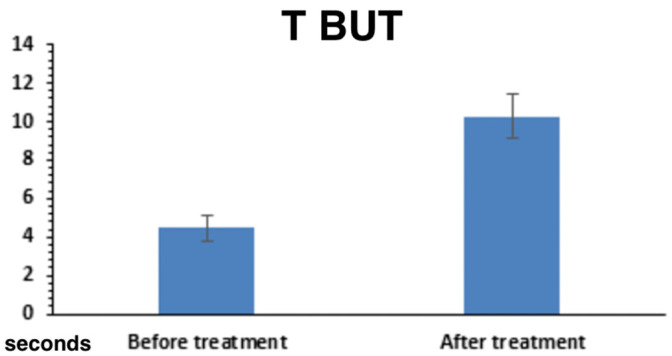
TBUT.

TBUT: 2.54 ± 0.62 s at baseline → 7.41 ± 0.57 s at final follow-up (*p* < 0.001) (Figure 4).

Schirmer I: progressive increases across groups (*p* < 0.05).

These changes indicate not only epithelial recovery but also restoration of tear film function.

#### 2.3.2. Corneal Epithelial Integrity (Oxford Grading)

**Figure 5:**
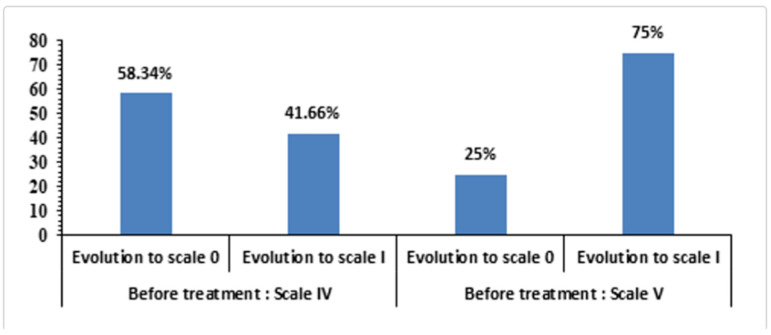
Oxford grading scale.

**Figure 6:**
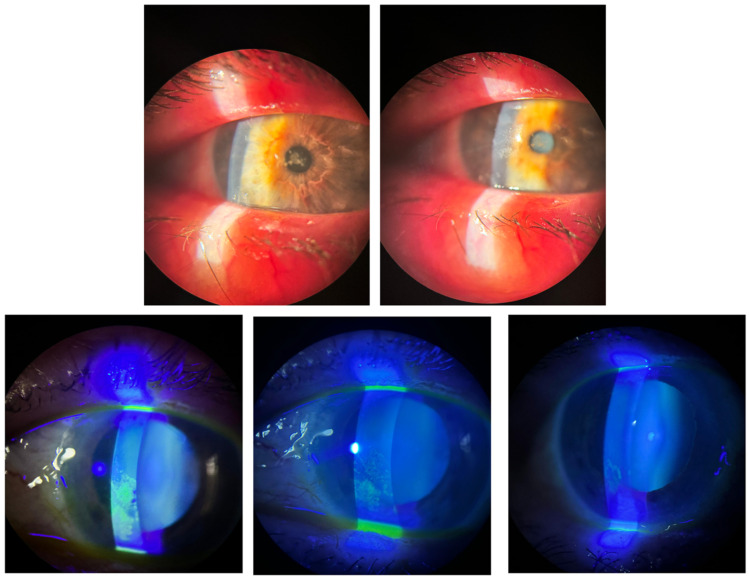
A representative case from group I before and after the treatment: the top 2 images are of a patient with rheumatologic disease and a filamentary keratitis; the bottom three images are of an epithelial defect in a patient with rheumatologic disease before the treatment (on the **left**), after one month (on the **center**) and after 2 months (on the **right**).

**Figure 7:**
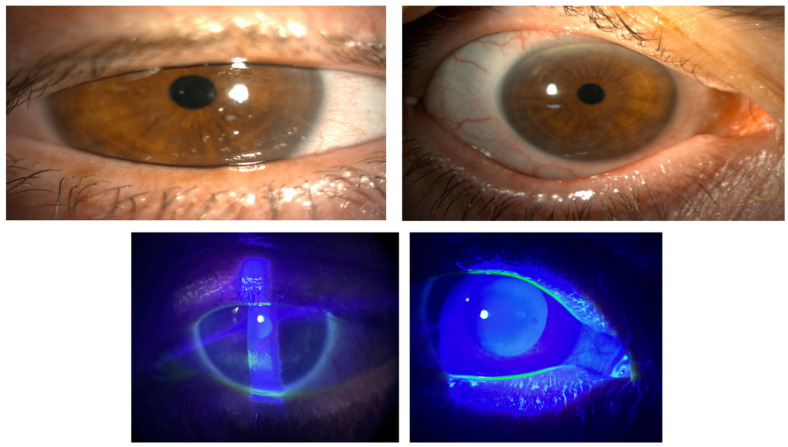
A representative case from group I before (figures on the **left**) and after the treatment (figures on the **right**).

At entry, all patients had Oxford grade IV or V:

From grade IV, 58.34% improved to grade 0 and 41.7% to grade I; from grade V, 25% reached grade 0 and 75% improved to grade I (Figure 5). Representative slit-lamp images (Figure 6 and Figure 7) show reduction in staining and closure of epithelial defects, particularly in Groups I–II.

#### 2.3.3. Best-Corrected Visual Acuity (BCVA)

By the end of the study, 43.8% of patients gained ≥2 ETDRS lines, with overall improvement vs. baseline (*p* < 0.05). Gains were greatest where vision loss reflected active epithelial disease rather than irreversible stromal/neuropathic changes.

### 2.4. Outcomes in Group III: Neurotrophic Corneal Ulcers

**Figure 8:**
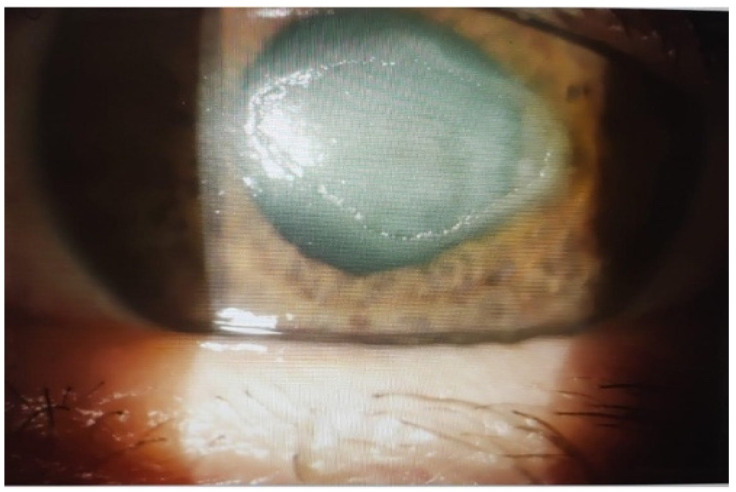
A representative case from group III: neurotrophic ulcer.

Among 10 patients with neurotrophic ulcers (Figure 8): complete healing in 7/10 (70%); partial healing (≥50% reduction) in 2/10 (20%); minimal response in 1/10 (10%). Ulcer area reduction was significant from Day 5 and continued through Day 30, 60, and 90 (*p* < 0.001). This trajectory is consistent with the trophic milieu of UCBS (e.g., NGF) supporting regeneration in denervated cornea.

### 2.5. Safety Profile

No treatment-related adverse events occurred. One case of bacterial conjunctivitis (unrelated to UCBS) was resolved with standard therapy. The overall safety signal is consistent with prior UCBS experience and quality-controlled preparation [19,20].

## 3. Discussion

Severe, refractory DED and related ocular surface disorders remain difficult to stabilize despite guideline-based care and escalation to immunomodulatory therapy [3,7]. In this cohort—strictly composed of non-responders—UCBS produced clinically meaningful gains across patient-reported outcomes (OSDI, SANDE, VAS) and objective metrics (TBUT, Schirmer, Oxford staining, BCVA) [21]. These findings align with the biological rationale for UCBS, which delivers a repertoire of epitheliotrophic and anti-inflammatory factors relevant to epithelial closure, barrier restoration, and pain control.

### 3.1. Relationship to Prior Evidence

Blood-derived therapies have long been used to support ocular surface repair. Autologous serum improves signs and symptoms in many patients but requires venipuncture and may carry pro-inflammatory mediators in active autoimmune disease [17]. UCBS, by contrast, is readily sourced at birth and contains higher concentrations of EGF, NGF, TGF-β, and VEGF, coupled with an anti-inflammatory cytokine profile [13]. Prior clinical studies reported improvements in severe DED and GVHD, with some suggesting superiority over autologous serum for corneal staining and goblet-cell metrics, particularly in Sjögren’s syndrome [16]. Our results are consistent with that signal: rapid symptom relief, faster epithelial recovery, and no treatment-related adverse events.

### 3.2. Clinical Impact by Etiology

The therapeutic effect was broad, with variation in magnitude by underlying disease:

- Rheumatologic disease (Group I). Patients with Sjögren’s/systemic sclerosis typically present with profound aqueous deficiency and persistent epithelial compromise [21,22]. In our series, 85.6% achieved complete epithelial healing within one week despite prior failures with cyclosporine or therapeutic lenses.
- Ocular GVHD (Group II). GVHD disrupts multiple components of the ocular surface system, including meibomian gland function, with marked tear film instability [22]. We observed substantial reductions in redness, pain, and staining, in line with earlier UCBS reports in GVHD.
- Neurotrophic ulcers (Group III). Given the role of NGF and other peptides in corneal nerve repair, the high ulcer-healing rate observed here is biologically coherent and mirrors early findings in related trophic strategies [23,24].
- Stevens–Johnson syndrome (Group IV). Although under-represented, SJS cases tolerated UCBS well, with improved epithelial stability and no safety signals.

### 3.3. Safety Profile

No treatment-related local or systemic adverse events were recorded. One case of bacterial conjunctivitis, deemed unrelated to UCBS, resolved with standard therapy. This aligns with published safety experience for UCBS and with quality-controlled processing frameworks. As always, rigorous donor screening and standardized preparation are essential to minimize infection risk and reduce variability in growth-factor content across batches [19,20].

### 3.4. Study Limitations

This was a retrospective, non-comparative analysis, which limits causal inference. Subgroup sizes were uneven—particularly SJS—and follow-up was ~4 months, precluding conclusions on long-term durability. We did not perform head-to-head comparisons with autologous serum or platelet-based preparations; therefore, relative efficacy cannot be inferred.

### 3.5. Future Directions

Priorities include randomized controlled trials comparing UCBS with autologous serum and recombinant growth-factor approaches; harmonized protocols for donor screening, processing, concentration, dosing, and storage; and prospective studies powered by etiology-specific outcomes. Given its enriched trophic profile, umbilical cord blood platelet lysate merits dedicated evaluation in non-healing ulcers and other refractory phenotypes [25,26].

## 4. Materials and Methods

### 4.1. Study Design and Oversight

We conducted a retrospective, interventional, non-randomized study at the Eye Center “G.B. Morgagni-DSV” (Catania, Italy) from May 2021 to May 2024. All procedures complied with the Declaration of Helsinki and good clinical practice. Umbilical Cord Blood Eyedrops therapy falls within the essential levels of care, and it is recommended by Italian Law, which ensures procedures controlled by the Ministry of Health. The Institutional Review Board and Ethics Committee of the Università degli Studi di Enna “Kore” was informed, which confirmed that approval was not needed for treating patients with routine and approved standard care. Written informed consent was obtained from all adult participants; for minors, consent was obtained from parents or legal guardians. All patients provided written informed consent to the use of their anonymized data for research purposes and scientific publications. The use of data collected from this study to prepare this study and publish the results was approved by the Institutional Review Board of dell’Università degli Studi di Enna “Kore” (protocol code 15395, 25 July 2025).

### 4.2. Participants and Clinical Groups

We included 55 patients (35 women, 20 men; 18–82 years) with severe DED or complex ocular surface disorders refractory to prior standard care (preservative-free lubricants, topical corticosteroids, and/or immunomodulators). Patients were assigned to four etiologic groups:

- Group I (n = 26): filamentary keratitis and/or corneal ulcers associated with systemic rheumatologic disease (e.g., Sjögren’s syndrome, systemic sclerosis)
- Group II (n = 15): ocular graft-versus-host disease (GVHD)
- Group III (n = 10): neurotrophic corneal ulcers.
- Group IV (n = 4): Stevens–Johnson syndrome (SJS).

Inclusion criteria: severe symptoms/signs at baseline (high VAS/SANDE/OSDI; advanced Oxford grade) and documented failure of prior therapy. Exclusion criteria: active ocular infection requiring immediate antimicrobial monotherapy or inability to complete follow-up.

Prior to the commencement of UCBS eyedrop therapy, all patients had undergone conventional therapies, which included preservative-free lubricants, topical corticosteroids, and cyclosporine 0.05%. The therapies were terminated at least seven days prior to the beginning of UCBS to guarantee a washout period.

### 4.3. Preparation of Allogeneic Umbilical Cord Blood Serum (UCBS)

Umbilical cord blood was collected immediately after vaginal or cesarean delivery following maternal consent. Up to 250 mL was obtained via the umbilical vein, allowed to clot at room temperature for 2 h, and centrifuged for 15 min. Serum was separated under sterile conditions, diluted to 20% with sterile isotonic solution, and dispensed into UV-protected sterile vials. Unopened vials were stored at −20 °C (≤3 months); opened vials were kept at +4 °C. All donations underwent microbiological/serologic screening (HBV, HCV, HIV, syphilis, toxoplasma, CMV) with batch traceability. Processing and storage parameters align with published sterility and growth-factor stability guidance [19,20].

### 4.4. Treatment Regimen

UCBS 20% was instilled one drop six times daily in the affected eye(s), generally for up to 90 days, with adjustments based on clinical response at follow-up.

### 4.5. Examinations and Outcome Measures

Assessments were performed at baseline (Day 0), Day 5, Day 30, Day 60, and the final visit (~4 months):

- Symptoms: Ocular Surface Disease Index (OSDI); SANDE frequency and severity; 10 cm visual analog scale (VAS, 0–100 mm; mean of seven symptom items).
- Ocular surface integrity: fluorescein staining graded by Oxford classification with slit-lamp photography; lissamine green when indicated.
- Tear film: non-invasive break-up time (NIBUT, keratography/CSO) and fluorescein TBUT.
- Visual function: best-corrected visual acuity (BCVA, ETDRS).
- Lacrimation: Schirmer I test (no anesthesia).
- Meibomian glands: meibography (gland dropout; lid margin changes including Marx line.
- Corneal sensitivity: esthesiometry when applicable.
- Clinician-rated signs: corneal inflammation, conjunctivalization, corneal neovascularization, pain.
- Ulcer subgroup (Group III): ulcer area and percent reduction versus baseline recorded at each visit.

### 4.6. Statistical Analysis

Analyses were performed with IBM SPSS v20.0. Quantitative variables are reported as mean ± SD; distribution was assessed by Shapiro–Wilk and histogram inspection. Pre/post comparisons used paired Student’s t-tests. Qualitative variables are counts and percentages. Two-sided *p* < 0.05 was considered statistically significant.

### 4.7. Composition of Umbilical Cord Blood Serum Eyedrops

From a biochemical point of view, like other hemoderivatives, umbilical cord blood shares several aspects with physiological film tear.

First of all, umbilical cord blood serum and tears share the same pH (7.4) and the same osmolality (around 280–300 mOsm/kg H_2_O), avoiding shrinkage (hypertonic effect) or swelling (hypotonic effect) on conjunctival and corneal cells and maintaining tight-junctions integrity [10,12].

UCBS contains a spectrum of epitheliotrophic growth factors that are also present in tears, but with significantly higher concentrations [27]:

- Epidermal Growth Factor (EGF): It is a mitogen, promotes cell growth and differentiation, and helps corneal heal wounds. It is present in a concentration of 0.2–0.3 ng/mL in tears and 0.5 ng/mL in UCBS.
- Transforming Growth Factor-beta (TGF-β)—It is a pleiotropic effect molecule and plays a key role in cellular processes such as cell growth, cell differentiation, and apoptosis. It is present in a concentration of 2–10 ng/mL in tears and 6–33 ng/mL in UCBS.
- Nerve Growth Factor (NGF)—Critical for the survival, maintenance and repair of corneal nerves. Surprisingly, its concentration is significantly lower in UCBS (54 pg/mL) than in tears (468 pg/mL)
- Insulin-like Growth Factor-1(IGF-1)—Involved in promoting keratinocytes growth and enhance their synthesis of collagens and other components of extracellular matrix. It is present in a concentration of 0.3 ng/mL in tears and 105 ng/mL in UCBS.
- Platelet-derived growth factor (PDGF): it promotes epithelial and stromal cell proliferation on the ocular surface, migration, and wound healing by stimulating fibroblast activity and extracellular matrix remodeling. It is present in a concentration of 1.3 ng/mL in tears and 15.4 ng/mL in UCBS [12].

**Figure 9:**
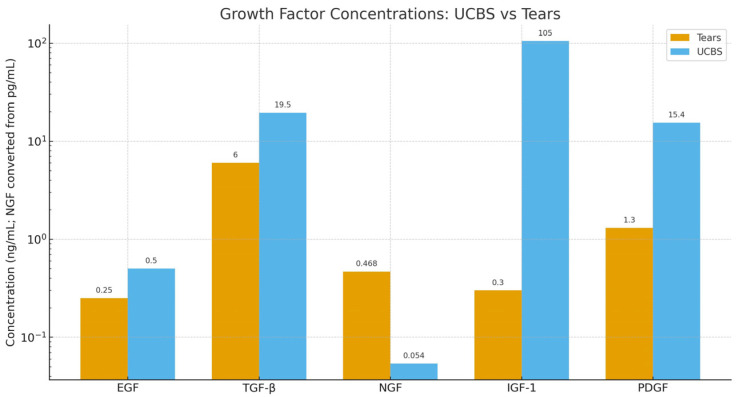
Comparison of growth factors in tears and UCBS using a logarithmic scale.

Growth factors are not the only biologically effective molecules contained in UCBS: albumin, vitamin A, fibronectin, and lactoferrin act as complementary trophic and defensive agents for the ocular surface. Albumin (0.023 mg/mL in tears and 53 mg/mL in UCBS) serves as a carrier and antioxidant buffer, binding free fatty acids, heme, and reactive aldehydes, thereby limiting oxidative and proteolytic damage while helping maintain osmotic balance and tear film stability. Vitamin A (0.02 mg/mL in tears and 46 mg/mL in UCBS) drives epithelial differentiation through RAR/RXR signaling, preserves conjunctival goblet cells, promotes mucin expression and prevents keratinization [14]. Figure 9 shows growth factors in tears compared to those present in UCBS.

UCBS eyedrops show significant anti-inflammatory effect that helps to alleviate symptoms of Dry Eye Disease: it is well-known that in severe DED, there is a prevalence of pro-inflammatory cytokines such as IL-1 and IL-6, which perpetuate epithelial damage, reduce the potential for corneal repair, and worsen the patient’s symptoms [2].

**Figure 10:**
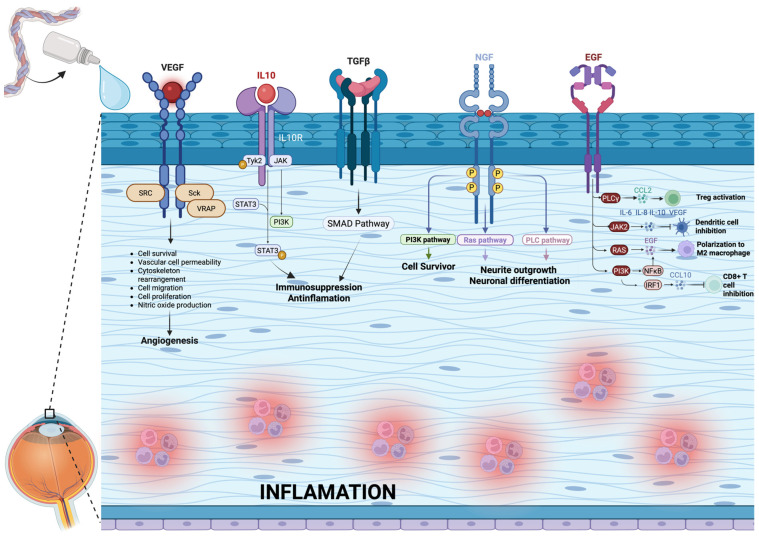
Mechanisms and factors involved in the inflammation of the ocular surface. Created in BioRender. Avitabile, A. (2025) https://BioRender.com/f9havfd.

The anti-inflammatory activity of UBCS can be attributed to its cytokine profile, high levels of anti-inflammatory molecules, such as IL-10, while pro-inflammatory cytokines like IL-1 and TNF are present at minimal concentrations [13]. Figure 10 shows the different mechanisms and factors involved in inflammation of the ocular surface.

## 5. Conclusions

In a cohort of patients with severe, treatment-refractory DED and complex ocular surface disease, UCBS eyedrops were well-tolerated and associated with substantial improvements in symptoms (OSDI, SANDE, VAS) and objective signs (TBUT, Schirmer I, Oxford staining, BCVA). These results support UCBS as a credible adjunct or rescue therapy when conventional approaches fail.

From a translational standpoint, earlier consideration of UCBS may be warranted in phenotypes driven by epithelial instability and inflammation. To solidify its role, the field now needs comparative trials against autologous serum and standardized protocols that reduce between-center variability in product composition and dosing.

## Figures and Tables

**Table 1:** Distribution of patients and key characteristics of study groups.

Group	No. of Patients (Eyes)	Primary Clinical Condition	Key Characteristics
I—Rheumatologic diseases	26	Sjögren’s syndrome, systemic sclerosis, other connective tissue diseases	Severe aqueous tear deficiency with persistent epithelial defects, pain, and photophobia. All had previously failed treatment with cyclosporine and therapeutic contact lenses.
II—Ocular GVHD	15	Ocular graft-versus-host disease following hematopoietic stem cell transplantation	Marked tear film instability with MGD, hyperemia, severe pain, and high risk of corneal ulceration.
III—Neurotrophic corneal ulcers	10	Corneal ulcers due to loss of corneal innervation	Poor healing response due to reduced corneal sensitivity, with high risk of perforation and irreversible visual loss.
IV—Stevens–Johnson Syndrome	4	Chronic sequelae of SJS/TEN	Profound epithelial instability and chronic inflammation with minimal response to conventional therapies.

**Table 2:** Pre- and post-treatment outcomes with UCBS eyedrops.

Outcome	Baseline (Mean ± SD)	Final Follow-Up (Mean ± SD)	*p* Value
OSDI (0–100) ↑ worse	89.57 ± 7.79	15.22 ± 11.33	<0.001
SANDE—Frequency (0–100) ↑ worse	87.83 ± 9.27	13.48 ± 8.85	<0.001
SANDE—Severity (0–100) ↑ worse	89.57 ± 7.79	15.22 ± 11.33	<0.001
VAS (0–100 mm) ↑ worse	88.40 ± 8.90	18.70 ± 9.10	<0.001
TBUT (seconds) ↑ better	2.54 ± 0.62	7.41 ± 0.57	<0.001
Schirmer I (mm/5 min) ↑ better	3.9 ± 1.1	8.6 ± 1.7	<0.001

## Data Availability

The original contributions presented in this study are included in the article. Further inquiries can be directed to the corresponding author.
